# Impact of Acute Kidney Injury on Mortality Outcomes in Patients Hospitalized for COPD Exacerbation: A National Inpatient Sample Analysis

**DOI:** 10.3390/jcm14155393

**Published:** 2025-07-31

**Authors:** Zeina Morcos, Rachel Daniel, Mazen Hassan, Hamza Qandil, Chloe Lahoud, Chapman Wei, Suzanne El Sayegh

**Affiliations:** 1 Internal Medicine, Staten Island University Hospital, Staten Island, NY 10305, USA; rdaniel4@northwell.edu (R.D.); mhassan14@northwell.edu (M.H.); hqandil@northwell.edu (H.Q.); chloelahoud@gmail.com (C.L.); cwei4@northwell.edu (C.W.); 2 Department of Nephrology, Staten Island University Hospital, Staten Island, NY 10305, USA; selsayegh@northwell.edu

**Keywords:** acute kidney injury, COPD, COPD exacerbation, mortality, intubation, respiratory failure, vasopressor use, National Inpatient Sample (NIS)

## Abstract

**Background/Objectives:** Acute kidney injury (AKI) worsens outcomes in COPD exacerbation (COPDe), yet limited data compare the demographics and mortality risk factors of COPDe admissions with and without AKI. Understanding this association may enhance risk stratification and management strategies. The aim of this study was to identify demographic differences and mortality risk factors in COPDe admissions with and without AKI. **Methods:** We conducted a retrospective cohort study using the National Inpatient Sample (NIS) from 1 January 2016 to 1 January 2021. Patients aged ≥ 35 years with a history of smoking and a diagnosis of COPDe were included. Patients with CKD stage 5, end-stage kidney disease (ESKD), heart failure decompensation, urinary tract infections, myocardial infarction, alpha-1 antitrypsin deficiency, or active COVID-19 infection were excluded. Baseline demographics were analyzed using descriptive statistics. Multivariate logistic regression analysis was used to measure the odds ratio (OR) of mortality. Statistical analyses were conducted using IBM SPSS Statistics V.30, with statistical significance at *p* < 0.05. **Results:** Among 405,845 hospitalized COPDe patients, 13.6% had AKI. These patients were older, had longer hospital stays, and included fewer females and White patients. AKI was associated with significantly higher mortality (OR: 2.417), more frequent acute respiratory failure (OR: 4.559), intubation (OR: 10.262), and vasopressor use (OR: 2.736). CVA, pneumonia, and pulmonary hypertension were significant mortality predictors. Hypertension, CAD, and diabetes were associated with lower mortality. **Conclusions:** AKI in COPDe admissions is associated with worse outcomes. Protective effects from certain comorbidities may relate to renoprotective medications. Study limitations include coding errors and retrospective design.

## 1. Introduction

A COPDe is defined as a worsening of symptoms, including a change in at least one of the following symptoms: increase in sputum volume, change in sputum character, increase in dyspnea, or change in cough [1]. A COPDe is a major healthcare challenge, often leading to hospitalizations due to the sudden worsening of symptoms. About 4.3% of all adults, in the United States of America, have been diagnosed with COPD [2]. Each year, COPDe leads to approximately 791,000 ED visits and over 700,000 hospitalizations [2,3]. One of the serious complications that can arise in these patients is acute kidney injury (AKI), which has been linked to worse outcomes, including higher mortality rates. AKI is defined per the Kidney Disease: Improving Global Outcomes (KDIGO) guidelines as a serum creatinine increase of ≥0.3 mg/dL (or ≥1.5 to 1.9 times baseline) or urine output < 0.5 mL/kg/h for 6–12 h [4]. However, there is limited understanding of the specific demographic factors and risk factors that contribute to higher mortality in patients with both COPD exacerbations and AKI. Previous research has demonstrated worsening outcomes in patients with both COPD and AKI, but further research is needed to pinpoint the specific demographics and clinical factors that increase mortality risk in this vulnerable population [5].

This study aims to fill that gap by analyzing a large national database to explore differences in demographics and identify key factors that increase the risk of mortality in patients hospitalized with COPDe, comparing those who develop AKI with those who do not. By better understanding these factors, we hope to improve the way we assess risks in these patients and help guide interventions that could reduce the negative impact of AKI on their health outcomes.

## 2. Materials and Methods

Study design and database: This retrospective cohort analysis was conducted using the Nationwide Inpatient Sample (NIS) database from 1 January 2016 until 1 January 2021. The NIS constitutes the largest inpatient care database in the United States. Institutional Review Board approval was deemed exempt under the Healthcare Cost and Utilization Project (HCUP) data use agreement (DUA) due to the de-identification of patient information. The research adhered to ethical guidelines set forth by the institution and HCUP-DUA.

Participants and eligibility criteria: Adult patients aged 35 years and older diagnosed with COPD and smokers were included. Patients were categorized into two groups, based on the presence of concomitant AKI at or during hospitalization. Patients were excluded if they had CKD stage 5, ESKD, acute heart failure exacerbation, acute myocardial infarction, acute urine infection, COVID-19 infection, or alfa one anti-trypsin deficiency. These criteria were chosen to focus the study on the specific impact of AKI on COPDe outcomes and to minimize potential confounding factors. Key data points included patient demographics (age, sex, race), comorbidities and complications such as acute respiratory failure, vasopressor use, and intubation. ICD-10 codes were used to identify diagnoses and clinical conditions within the National Inpatient Sample dataset.

Statistical analysis: Baseline characteristics were analyzed using descriptive statistics. Continuous variables were compared using an independent *t*-test (for parametric data) or the Mann–Whitney U test (for nonparametric data). In contrast, categorical variables were compared using the chi-square test or Fisher’s exact test. A multivariate logistic regression model was used to assess the independent effect of each variable on inpatient mortality. Odds ratios (ORs) with 95% confidence intervals (CIs) were calculated. All statistical analyses were performed using the Statistical Package for the Social Sciences (version 30; IBM Corp., Armonk, NY, USA). Statistical significance was set at P less than 0.05.

## 3. Results

### 3.1. Demographical Data

#### 3.1.1. Baseline Demographics

Among 405,845 hospitalized COPDe patients, 55,270 (13.6%) had concomitant AKI. Patients with AKI were older than those without AKI (mean age: 72.69 ± 10.29 vs. 69.55 ± 10.84 years, *p* = 0.01) and had a longer hospital stay (6.12 ± 5.40 vs. 4.58 ± 3.98 days, *p* = 0.015) (Table 1). The proportion of female patients was lower in the AKI group (44% vs. 54.2%, *p* < 0.001). Racial distribution varied significantly between groups (*p* < 0.001). A lower proportion of White patients had AKI (76.9% vs. 80.5%), while Black (13.9% vs. 11.4%) and Hispanic (5.3% vs. 4.8%) patients had higher AKI prevalence.

#### 3.1.2. Mortality and Inpatient Complication Demographics

Mortality was significantly higher in patients with AKI (5.8% vs. 1.6%, *p* < 0.001). Acute respiratory failure was more common (58.4% vs. 51.7%, *p* < 0.001), with a greater need for intubation (10.7% vs. 3.9%, *p* < 0.001) and vasopressor support (1.2% vs. 0.2%, *p* < 0.001), indicating a higher severity of COPDe with concomitant AKI. Additionally, pneumonia was more frequently diagnosed in the AKI group (42.2% vs. 35.4%, *p* < 0.001) (Table 1).

#### 3.1.3. Comorbidity Distribution

Patients with AKI had a significantly higher prevalence of comorbidities compared to those without AKI (Table 1). Hypertension was the most prevalent condition, affecting 84.3% of patients with AKI versus 72.4% of those without (*p* < 0.001). Similarly, diabetes mellitus (40.3% vs. 28.7%, *p* < 0.001) and dyslipidemia (36.5% vs. 29.4%, *p* < 0.001) were more common in the AKI group. Chronic kidney disease (CKD) stage 3–4 was notably higher among AKI patients (31% vs. 7.1%, *p* < 0.001), as was coronary artery disease (CAD) (35% vs. 27.7%, *p* < 0.001). Other comorbid conditions, including obstructive sleep apnea (OSA) (16.6% vs. 13.8%, *p* < 0.001), cerebrovascular disease (CVA) (0.7% vs. 0.4%, *p* < 0.001), and pulmonary hypertension (11.1% vs. 8.2%, *p* < 0.001), were also more prevalent in patients with AKI. However, gastroesophageal reflux disease (GERD) was slightly less prevalent among AKI patients (27.1% vs. 28.6%, *p* < 0.001). This pattern highlights a greater burden of cardiovascular, metabolic, and respiratory complications in patients with AKI, underscoring their increased vulnerability during COPDe hospitalizations.

### 3.2. Multivariate Analysis Data

#### 3.2.1. Demographic Risk Factors and Mortality

After adjusting for confounders, AKI was associated with a significantly higher risk of inpatient mortality (OR: 2.417, 95% CI: 2.295–2.545, *p* < 0.001) (Table 2). Older age also increased mortality risk (OR: 1.049, 95% CI: 1.046–1.051, *p* < 0.001), while female sex was associated with a lower mortality risk (OR: 0.872, 95% CI: 0.833–0.913, *p* < 0.001). Among racial groups, Black (OR: 0.718, 95% CI: 0.660–0.781, *p* < 0.001) and Hispanic (OR: 0.783, 95% CI: 0.697–0.879, *p* < 0.001) patients had lower odds of mortality compared to White patients.

#### 3.2.2. Hospital Complications and Mortality

**Figure 1:**
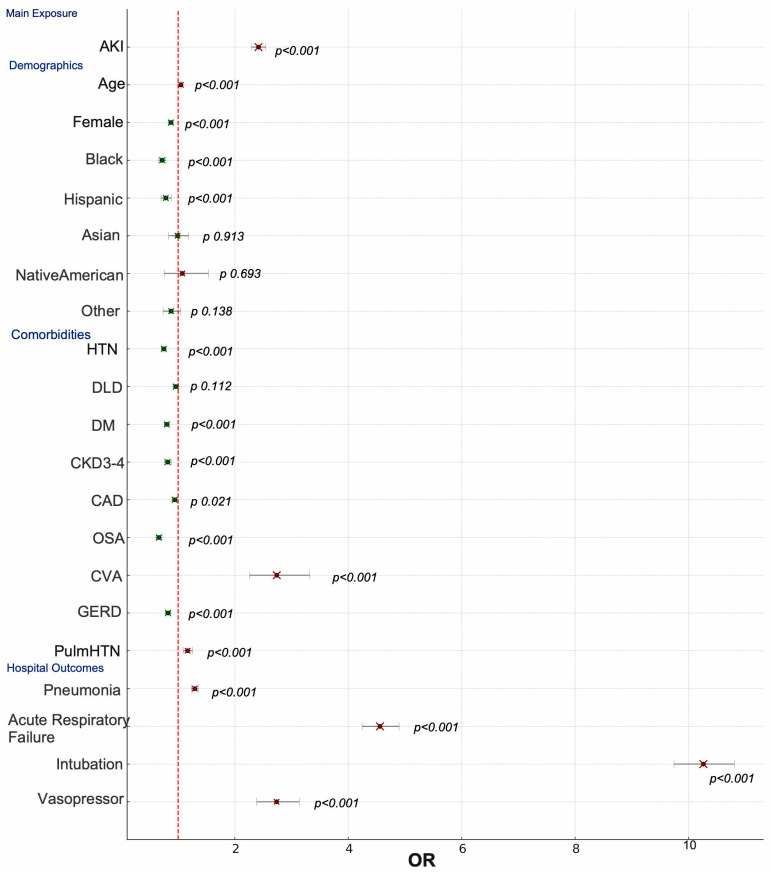
Forest plot of mortality risk factors in COPDe patients with AKI. HTN—hypertension; DLD—dyslipidemia; CKD—chronic kidney disease; CAD—coronary artery disease; OSA—obstructive sleep apnea; CVD—cerebral vascular disease; GERD—gastroesophageal reflux disease; Pulm HTN—pulmonary hypertension. The dashed vertical line represents the null value (OR = 1). Green square within the CI indicate variables associated with a protective effect (OR < 1) while the Red indicate variables associated with a higher mortality risk (OR > 1).

Moreover, intubation had the highest mortality risk (OR: 10.262, 95% CI: 9.744–10.808, *p* < 0.001). Acute respiratory failure was strongly associated with mortality (OR: 4.559, 95% CI: 4.245–4.897, *p* < 0.001), followed by vasopressor use (OR: 2.736, 95% CI: 2.385–3.138, *p* < 0.001). Moreover, pneumonia showed an OR of 1.293 (95% CI: 1.236–1.354, *p* < 0.001) (illustrated in Figure 1).

#### 3.2.3. Comorbidities and Mortality

Cerebrovascular disease (CVA) was another major risk factor (OR: 2.739, 95% CI: 2.259–3.321, *p* < 0.001), and pulmonary hypertension (OR: 1.167, 95% CI: 1.088–1.252, *p* < 0.001) was also a significant contributor (Table 2). Conversely, hypertension (OR: 0.747, 95% CI: 0.709–0.787, *p* < 0.001) and diabetes (OR: 0.800, 95% CI: 0.758–0.844, *p* < 0.001) were associated with lower mortality risk. CKD stage 3–4 also showed an OR of 0.816 (95% CI: 0.758–0.879, *p* < 0.001); OSA (OR: 0.660, 95% CI: 0.610–0.714, *p* < 0.001) and GERD (OR: 0.821, 95% CI: 0.778–0.867, *p* < 0.001) were also linked to reduced mortality. CAD had a modest protective effect (OR: 0.94, 95% CI: 0.893–0.991, *p* = 0.021). while DLD showed no statistically significant impact on mortality (OR: 0.959, 95% CI: 0.911–1.01, *p* = 0.112) (illustrated in Figure 1). These findings highlight that while cardiovascular and metabolic conditions such as hypertension and diabetes may be protective, CVA and pulmonary hypertension are key contributors to increased mortality risk in AKI patients hospitalized for COPDe. We hypothesize that the protective effect of hypertension, diabetes, and CAD in AKI patients may be due to the widespread use of renoprotective medications in this group, including ACE inhibitors, ARBs, and SGLT2 inhibitors.

## 4. Discussion

A chronic obstructive pulmonary disease exacerbation (COPDe) is an acute clinical event defined by a sudden and sustained worsening of respiratory symptoms, such as increased dyspnea, cough, and sputum production, in patients with an established history of COPD, often linked to a significant smoking background. These exacerbations contribute substantially to healthcare utilization, with frequent emergency department visits, hospital admissions, and increased morbidity and mortality. The clinical course of a COPDe can be further complicated by the development of acute kidney injury (AKI), an increasingly recognized comorbidity that adversely affects patient outcomes. AKI, which may arise due to hypoperfusion, nephrotoxic medications, or systemic inflammation, has been associated with increased mortality and prolonged recovery in various hospitalized populations, including those with pulmonary disease. Identifying AKI in the context of COPDe can serve as an important prognostic marker and may influence the aggressiveness of inpatient management.

In this retrospective cohort study, we sought to investigate the incidence and prevalence of AKI in patients hospitalized with COPDe and to evaluate its impact on key clinical outcomes, including inpatient mortality and hospital length of stay (LOS), in comparison to patients hospitalized with COPDe alone. The study population included adults aged 35 and older with a documented history of cigarette smoking and a confirmed diagnosis of COPD. Patients were categorized into two groups based on whether they developed AKI either at the time of admission or during the course of hospitalization.

To ensure a focused analysis, several exclusion criteria were applied, including patients with end-stage renal disease (ESRD), acute heart failure exacerbation, ongoing urinary tract infection (UTI), myocardial infarction, alpha-1 antitrypsin deficiency, and those with a concurrent COVID-19 diagnosis. These conditions were excluded due to their potential to confound the clinical picture and independently contribute to kidney injury or mortality.

The study’s findings revealed that patients who developed AKI during hospitalization for COPDe exhibited markedly worse clinical outcomes compared to those without AKI. Significant differences were observed in demographic profiles, presence of comorbidities, complexity of hospital course, longer LOS, and, notably, higher inpatient mortality rates. These results suggest that AKI serves not only as a complication but also as a potential marker of disease severity in the setting of COPD exacerbations.

Given these findings, early identification of patients at risk for AKI upon hospital admission may allow for more targeted interventions, including closer hemodynamic monitoring, judicious use of nephrotoxic medications, and timely nephrology consultation. Furthermore, these insights can aid in risk stratification, inform prognosis, and support the development of multidisciplinary care strategies aimed at improving outcomes in this high-risk population.

Our analysis revealed notable demographic differences between COPD patients with and without concomitant AKI. One key finding was the significant age difference between the two groups. Patients with AKI were generally older than those without, with a mean age of 72.69 ± 10.29 years compared to 69.55 ± 10.84 years in the non-AKI group (*p* = 0.01). This association may be explained by age-related declines in estimated glomerular filtration rate (eGFR), underscoring advanced age as a significant risk factor for AKI in patients with COPD, as shown by Singh et al. [6]. In addition, older patients are more likely to have pre-existing chronic kidney disease (CKD), which may result from underlying conditions such as renal artery disease, diabetic nephropathy, or longstanding hypertension—all of which increase susceptibility to AKI during hospitalization.

In addition to age, differences in sex and ethnicity were also observed. Females were found in lower proportion in the AKI group compared to the non-AKI group. This disparity may be attributed to several factors, including the protective effects of estrogen on both cardiovascular and renal function, which may help reduce the severity or incidence of AKI. Moreover, females generally have a lower prevalence of comorbid conditions such as coronary artery disease and chronic kidney disease. They are also more likely to seek medical attention earlier and adhere more consistently to treatment regimens, which can lead to improved management of both COPD and its complications, including AKI [7,8,9].

White patients were also found in lower proportion in the AKI group compared to the non-AKI group. This observation may suggest a lower incidence of kidney injury in this population. In contrast, Black and Hispanic patients were represented in greater proportion within the AKI group. These demographic differences raise important questions about racial and ethnic susceptibilities to AKI in the setting of COPD, warranting further investigation [7,9]. Grams et al. [10] suggest that socioeconomic disparities such as differences in income, education level, and insurance coverage may influence access to preventive care and timely medical interventions, potentially contributing to this trend.

As expected, patients with AKI exhibited a significantly higher prevalence of comorbidities compared to those without AKI. Hypertension was the most common condition, affecting 84.3% of patients in the AKI group versus 72.4% in the non-AKI group. Similarly, the rates of diabetes mellitus and dyslipidemia were also notably higher among patients with AKI. These findings align with the established literature and can be explained by several pathophysiological mechanisms. Hypertension is a well-known risk factor for AKI, as it can result in chronic kidney damage and reduced renal reserve, thereby increasing vulnerability to acute renal insults [11]. Diabetes mellitus, likewise, more prevalent in the AKI group, contributes to AKI risk through its deleterious effects on microvascular integrity and its association with diabetic nephropathy [11,12]. Dyslipidemia, through its role in promoting atherosclerosis and cardiovascular disease, can impair renal perfusion and further exacerbate kidney injury [13].

Given that chronic kidney disease (CKD) is a well-established risk factor for the development of AKI, Wan et al. [7] demonstrated a clear link between CKD stages 3–4 and increased AKI risk. It is therefore not surprising that CKD stage 3–4 was notably more prevalent among patients with AKI. In addition to CKD, other comorbid conditions, including coronary artery disease (CAD), obstructive sleep apnea (OSA), cerebrovascular disease (CVD), and pulmonary hypertension, were also more commonly seen in the AKI group. Interestingly, gastroesophageal reflux disease (GERD) was slightly less prevalent among patients with AKI. The presence of these comorbidities likely compounds physiological stress during a COPD exacerbation, increasing the risk for renal injury. These findings underscore the importance of comprehensive and proactive management of chronic comorbid conditions to reduce the risk of AKI and improve overall patient outcomes.

Patients hospitalized for COPD exacerbations often require advanced respiratory support to stabilize their oxygenation status, including high-flow nasal cannula, noninvasive ventilation (NIV), or mechanical ventilation. These interventions may prolong hospitalization, particularly when complications such as AKI arise. Our analysis revealed that patients who developed AKI during hospitalization for COPD exacerbation had significantly longer lengths of stay (LOSs), with a mean LOS of 6 days compared to 4 days for those without AKI. In a study by Zhang et al. [14], patients with acute COPD exacerbation complicated by AKI had substantially longer hospital stays (13 days vs. 10 days) and significantly higher in-hospital mortality (18.0% vs. 2.7%) than those without AKI.

These findings further emphasize the importance of early intervention to optimize oxygenation and avoid clinical deterioration. The extended LOS observed in the AKI group may be attributed to several factors, including respiratory failure, the need for close monitoring of fluid balance, correction of electrolyte abnormalities, and impaired metabolic compensation for respiratory acidosis. Identifying patients at higher risk for AKI and initiating timely, targeted interventions may help reduce hospitalization duration and improve survival.

Our data supports that patients who developed AKI during COPD exacerbations experienced significantly higher inpatient mortality compared to those without AKI (5.8% vs. 1.6%). The elevated mortality in this population is likely multifactorial, though several mechanisms are supported by the existing literature. One proposed explanation involves impaired metabolic compensation for respiratory acidosis. As shown by Liu et al. [15], AKI diminishes the kidneys’ ability to retain bicarbonate, a key buffer against elevated carbon dioxide levels in COPD patients. This impairment can worsen respiratory acidosis and lead to acute respiratory failure, requiring escalated oxygen support and increasing the risk of mortality. Our data supports this association, as patients with AKI experienced higher rates of acute respiratory failure (58.4% vs. 51.7%) and a greater need for intubation (10.7% vs. 3.9%). Chen et al. further demonstrated that the interaction between acute respiratory failure and AKI synergistically increases the risk of in-hospital mortality in patients with COPD exacerbations, emphasizing the importance of managing both complications concurrently [8].

AKI is also associated with fluid overload and electrolyte abnormalities, particularly hyperkalemia and hyponatremia. Hyperkalemia may lead to serious electrophysiological disturbances, including bradyarrhythmia and potentially fatal cardiac arrhythmias. In severe cases, this can precipitate cardiac arrest and necessitate immediate intervention with mechanical ventilation and vasopressor support [16]. Hyponatremia, especially when rapidly fluctuating, can contribute to cerebral edema and neurological impairment. This, in turn, may lead to respiratory muscle weakness, precipitating acute respiratory failure and the need for ventilatory support.

Additionally, the presence of AKI in COPD patients can amplify systemic inflammation and oxidative stress. Elevated levels of inflammatory markers such as high-sensitivity C-reactive protein (hs-CRP) have been associated with poor prognosis in this cohort [17,18]. This pro-inflammatory state increases the risk of multi-organ dysfunction, further worsening patient outcomes.

To further explore predictors of mortality, a multivariate analysis was conducted. The findings indicated that intubation carried the highest risk of mortality, followed by vasopressor use, both of which strongly correlate with the severity of respiratory failure. Vieira et al. found that the presence of AKI was associated with delayed weaning from mechanical ventilation in critically ill patients, potentially prolonging hospital stay and increasing complication rates [18]. Faubel and Edelstein [19] demonstrated that AKI contributes to respiratory failure through systemic inflammation and immune dysregulation, which can lead to multi-organ dysfunction, including lung injury. Legrand and Rossignol [20] further highlighted the role of increased vascular permeability and pulmonary edema due to AKI. In patients requiring mechanical ventilation, careful management of fluid status and electrolyte balance becomes critical. In severe AKI cases, renal replacement therapy (RRT) may be necessary, which introduces further complexity, including hemodynamic instability that often requires vasopressor support. These overlapping processes create a vicious cycle of AKI and respiratory failure, leading to prolonged mechanical ventilation, challenges in weaning, and increased mortality risk. In addition to its acute consequences, AKI has been shown to contribute to the progression of chronic kidney disease, highlighting the need for long-term renal monitoring in COPD patients who experience AKI during hospitalization [12].

Cerebrovascular disease (CVD) also emerged as a major mortality risk factor. The relationship between CVD and AKI is complex and bidirectional. CVD can precipitate AKI through mechanisms such as hypoperfusion during stroke or cardiogenic shock. Additionally, the use of contrast agents in imaging can cause contrast-induced nephropathy, as described by Modi et al. [19]. Conversely, Albeladi et al. [20] demonstrated that AKI is associated with increased risk of future cerebrovascular events due to inflammation, atherosclerosis, and endothelial dysfunction. Shared risk factors, including hypertension, diabetes, and dyslipidemia, likely contribute to the co-occurrence of these conditions.

Pulmonary hypertension was another significant risk factor. It is defined by a mean pulmonary artery pressure of ≥25 mm Hg at rest, as established in guidelines by Galiè et al. [21]. Kidney dysfunction is frequently observed in patients with pulmonary hypertension and serves as an independent predictor of mortality. Nickel et al. [22] proposed mechanisms linking pulmonary hypertension to AKI, including increased venous congestion, decreased cardiac output, and neurohormonal activation. AKI may exacerbate cardiovascular strain and systemic inflammation, both of which are associated with poorer prognosis in critically ill patients, as described by Legrand and Rossignol [17].

Notably, the detrimental impact of AKI is not limited to acute exacerbations of COPD; Wang et al. [23] found that even among patients hospitalized with stable, non-exacerbated COPD, the presence of AKI independently predicted increased in-hospital mortality and prolonged hospitalization. This finding reinforces the critical role of renal function in determining outcomes across the full spectrum of COPD severity. Similarly, Barakat et al. found that the presence of AKI was associated with increased morbidity and mortality in both stable COPD and during exacerbations, further emphasizing the critical role of renal function across the COPD spectrum [9].

Interestingly, despite being more prevalent in the AKI group, comorbidities such as hypertension and diabetes were associated with lower mortality. Similarly, CKD stage 3–4, OSA, and GERD were linked to reduced mortality, and CAD showed a modest protective effect. Dyslipidemia showed no significant impact on mortality. These findings may reflect the protective effects of certain medications, such as ACE inhibitors, ARBs, and SGLT2 inhibitors, commonly prescribed in patients with hypertension, diabetes, and CAD. These agents have demonstrated both renal and mortality benefits, as reported by Cardoso et al. [24] and van Vark et al. [25], which may explain the observed decrease in mortality risk.

Although Black and Hispanic patients were more likely to develop AKI, their overall mortality was lower. Kabarriti et al. [26] suggest that these groups often have higher rates of hypertension and diabetes, leading to more frequent use of renoprotective therapies with known mortality benefits. Thus, increased exposure to these medications may help mitigate the elevated mortality risk typically associated with AKI. Additionally, disparities in hospitalization thresholds, access to care, and socioeconomic determinants may also play a role and warrant further investigation.

As a retrospective study, this analysis has several limitations. While it identifies associations between AKI and mortality in COPD exacerbations, it does not establish causality. Determining the temporal sequence of clinical events is difficult in retrospective datasets, and coding inaccuracies may confound the findings. The lack of longitudinal follow-up data also limits the ability to assess long-term outcomes and refine risk stratification tools. Additionally, potential selection and confounding biases may limit the generalizability of results to the broader COPD population.

Future research should focus on prospective, longitudinal studies to better characterize the temporal and causal relationships between AKI and outcomes in COPD exacerbations. Such studies should incorporate detailed clinical and biochemical data to improve risk prediction models and identify modifiable risk factors. Further exploration into the protective effects of commonly used medications could provide valuable insights into optimizing therapy. Additionally, addressing demographic disparities potentially driven by socioeconomic factors, comorbidity burden, and access to care will be critical for improving outcomes in vulnerable patient populations.

## 5. Conclusions

Finally, this study highlights the significant clinical impact of acute kidney injury in patients hospitalized for COPDe. AKI was associated with increased length of stay, higher rates of respiratory failure, greater need for mechanical ventilation and vasopressor support, and, notably, higher inpatient mortality. The presence of comorbidities such as cerebrovascular disease and pulmonary hypertension further compounded the risk of poor outcomes. While traditionally high-risk conditions like hypertension, diabetes, and CKD appeared protective in mortality models, this paradox may reflect the beneficial role of the renoprotective therapies frequently prescribed in these populations. Furthermore, racial disparities in AKI incidence and mortality suggest that medication access, disease management, and social determinants of health play critical roles in patient outcomes.

Indeed, early identification of at-risk patients, optimization of fluid and electrolyte management, and consideration of renoprotective strategies may help mitigate adverse outcomes. Future prospective studies are essential to establish causality, refine risk prediction, and develop targeted interventions that improve survival in this high-risk population.

## Figures and Tables

**Table 1:** Baseline characteristics comorbidities and outcomes among patients with acute COPDe (*n* = 405,913) with and without AKI during hospitalization.

Variable *	With AKI (*n* = 55,270)	Without AKI (*n* = 350,575)	*p*-Value
Age, mean (SD)	72.69 (±10.29)	69.55 (±10.84)	0.01 **
LOS, mean (SD)	6.12 (±5.40)	4.58 (±3.98)	0.015 ***
Female, *n* (%)	24,306 (44.0%)	190,102 (54.2%)	<0.001 ****
Race, *n* (%)			<0.001 ****
White	41,523 (76.9%)	275,654 (80.5%)	
Black	7515 (13.9%)	38,915 (11.4%)	
Hispanic	2880 (5.3%)	16,399 (4.8%)	
Asian	897 (1.7%)	4082 (1.2%)	
Native American	232 (0.4%)	1554 (0.5%)	
Other	981 (1.8%)	5894 (1.7%)	
Comorbidities, *n* (%)			
HTN	46,596 (84.3%)	253,850 (72.4%)	<0.001 ****
DLD	20,164 (36.5%)	103,124 (29.4%)	<0.001 ****
DM	22,274 (40.3%)	100,466 (28.7%)	<0.001 ****
CKD3–4	17,129 (31.0%)	24,920 (7.1%)	<0.001 ****
CAD	19,329 (35.0%)	97,045 (27.7%)	<0.001 ****
OSA	9191 (16.6%)	48,413 (13.8%)	<0.001 ****
CVD	412 (0.7%)	1314 (0.4%)	<0.001 ****
GERD	14,965 (27.1%)	100,214 (28.6%)	<0.001 ****
Pulm HTN	6143 (11.1%)	28,861 (8.2%)	<0.001 ****
Hospital outcomes, *n* (%)			
Pneumonia	23,326 (42.2%)	124,112 (35.4%)	<0.001 ****
Acute respiratory failure	32,268 (58.4%)	181,124 (51.7%)	<0.001 ****
Intubation	5918 (10.7%)	13,837 (3.9%)	<0.001 ****
Vasopressor support	651 (1.2%)	744 (0.2%)	<0.001 ****
Inpatient mortality	3213 (5.8%)	5728 (1.6%)	<0.001 ****

* HTN—hypertension; DLD—dyslipidemia; CKD—chronic kidney disease; CAD—coronary artery disease; OSA—obstructive sleep apnea; CVD—cerebral vascular disease; GERD—gastroesophageal reflux disease; Pulm HTN—pulmonary hypertension. ** Independent *t*-test. *** Mann–Whitney U test. **** Chi-square test.

**Table 2:** Multivariate logistic regression analysis for inpatient mortality among patients with acute COPDe and AKI.

Variable *	OR	95% CI (Lower)	95% CI (Upper)	*p*-Value
AKI	2.417	2.295	2.545	<0.001
Age	1.049	1.046	1.051	<0.001
Female	0.872	0.833	0.913	<0.001
Race (White = reference)				
Black	0.718	0.66	0.781	<0.001
Hispanic	0.783	0.697	0.879	<0.001
Asian	0.99	0.83	1.181	0.913
Native American	1.074	0.752	1.535	0.693
Other	0.876	0.735	1.044	0.138
Comorbidities				
HTN	0.747	0.709	0.787	<0.001
DLD	0.959	0.911	1.01	0.112
DM	0.8	0.758	0.844	<0.001
CKD3–4	0.816	0.758	0.879	<0.001
CAD	0.94	0.893	0.991	0.021
OSA	0.66	0.61	0.714	<0.001
CVD	2.739	2.259	3.321	<0.001
GERD	0.821	0.778	0.867	<0.001
Pulm HTN	1.167	1.088	1.252	<0.001
Hospital outcomes				
Pneumonia	1.293	1.236	1.354	<0.001
Acute respiratory failure	4.559	4.245	4.897	<0.001
Intubation	10.262	9.744	10.808	<0.001
Vasopressor	2.736	2.385	3.138	<0.001

* HTN—hypertension; DLD—dyslipidemia; CKD—chronic kidney disease; CAD—coronary artery disease; OSA—obstructive sleep apnea; CVD—cerebral vascular disease; GERD—gastroesophageal reflux disease; Pulm HTN—pulmonary hypertension.

## Data Availability

The data that support the findings of this study are available from the Healthcare Cost and Utilization Project (HCUP) National Inpatient Sample (NIS) database. Restrictions apply to the availability of these data, which were used under license. Data are available with permission from HCUP at: https://www.hcup-us.ahrq.gov/.

## Abbreviations

The following abbreviations are used in this manuscript:

AKI	Acute Kidney Injury
COPD	Chronic Obstructive Pulmonary Disease
COPDe	COPD Exacerbation
NIS	National Inpatient Sample
HCUP	Healthcare Cost and Utilization Project
LOS	Length of Stay
HTN	Hypertension
DLD	Dyslipidemia
DM	Diabetes Mellitus
CKD3–4	Chronic Kidney Disease Stage 3 to 4
CAD	Coronary Artery Disease
OSA	Obstructive Sleep Apnea
CVD	Cerebrovascular Disease
GERD	Gastroesophageal Reflux Disease
Pulm HTN	Pulmonary Hypertension
OR	Odds Ratio
CI	Confidence Interval
ESKD	End-Stage Kidney Disease
RRT	Renal Replacement Therapy
UTI	Urinary Tract Infection
KDIGO	Kidney Disease Improving Global Outcomes
